# Inference of Subpathway Activity Profiles Reveals Metabolism Abnormal Subpathway Regions in Glioblastoma Multiforme

**DOI:** 10.3389/fonc.2020.01549

**Published:** 2020-09-11

**Authors:** Xudong Han, Donghua Wang, Ping Zhao, Chonghui Liu, Yue Hao, Lulu Chang, Jiarui Zhao, Wei Zhao, Lili Mu, Jinghua Wang, Hulun Li, Qingfei Kong, Junwei Han

**Affiliations:** ^1^Department of Neurobiology, Harbin Medical University, Heilongjiang Provincial Key Laboratory of Neurobiology, Harbin, China; ^2^Department of General Surgery, General Hospital of Heilongjiang Province Land Reclamation Bureau, Harbin, China; ^3^Key Laboratory of Preservation of Human Genetic Resources and Disease Control in China (Harbin Medical University), Ministry of Education, Harbin, China; ^4^College of Bioinformatics Science and Technology, Harbin Medical University, Harbin, China

**Keywords:** GBM, gene, metabolic subpathway, steroid biosynthesis, U87-MG cell, (S)-2,3-Epoxysqualene

## Abstract

Glioblastoma, also known as glioblastoma multiforme (GBM), is the most malignant form of glioma and represents 81% of malignant brain and central nervous system (CNS) tumors. Like most cancers, GBM causes metabolic recombination to promote cell survival, proliferation, and invasion of cancer cells. In this study, we propose a method for constructing the metabolic subpathway activity score matrix to accurately identify abnormal targets of GBM metabolism. By integrating gene expression data from different sequencing methods, our method identified 25 metabolic subpathways that were significantly abnormal in the GBM patient population, and most of these subpathways have been reported to have an effect on GBM. Through the analysis of 25 GBM-related metabolic subpathways, we found that (S)-2,3-Epoxysqualene, which was at the central region of the sterol biosynthesis subpathway, may have a greater impact on the entire pathway, suggesting a potential high association with GBM. Analysis of CCK8 cell activity indicated that (S)-2,3-Epoxysqualene can indeed inhibit the activity of U87-MG cells. By flow cytometry, we demonstrated that (S)-2,3-Epoxysqualene not only arrested the U87-MG cell cycle in the G0/G1 phase but also induced cell apoptosis. These results confirm the reliability of our proposed metabolic subpathway identification method and suggest that (S)-2,3-Epoxysqualene has potential therapeutic value for GBM. In order to make the method more broadly applicable, we have developed an R system package crmSubpathway to perform disease-related metabolic subpathway identification and it is freely available on the GitHub (https://github.com/hanjunwei-lab/crmSubpathway).

## Introduction

The incidence of GBM accounts for 65% of all types of gliomas, and glioblastoma is the highest grade of glioma according to the World Health Organization (WHO) classification. GBM causes many changes in the cellular molecular mechanisms (1–3). For example, like most cancers, altered cellular metabolism is a hallmark of GBM. GBM rewires metabolism for numerous pro-growth and pro-survival functions, including macromolecule synthesis, ATP generation, and antioxidant regeneration. Hence, GBM metabolism is an area of intense research aiming to identify novel therapeutic targets and biomarkers (4).

At present, the analysis of GBM metabolism is basically dependent on experiments. These methods are not only long term but also costly. However, with the popularity and reduced cost of high-throughput sequencing technologies, high-throughput sequencing data related to malignant gliomas continue to emerge. How to use these data to comprehensively demonstrate the changes involved in the disease mechanism in a cost-effective manner has become a popular research direction (5). Currently, through the application of data mining algorithms in biology, genes associated with GBM are constantly being discovered (e.g., *COL3A1, FN1*, and *MMP9*) (6). However, limitations, such as poor stability and the lack of consideration of the biological relationships between genes, make the analysis at the genetic level questionable. Therefore, methods and tools for identifying pathways have been developed. For example, ctPath identifies differentially expressed pathways via demixing pathway crosstalk effect from transcriptomics data (7). ESEA (Edge Set Enrichment Analysis) identifies dysregulated pathways by investigating the changes in biological relationships of pathways in the context of gene expression data (8). However, even the smallest pathway still contains at least tens to hundreds of genes and these traditional pathway identification methods do not accurately consider the combined effect of the interesting molecules and neglects expression correlations or topological features embedded in the pathways (9). In the disease state, not all genes in the pathway are dysfunctional; Instead, genes usually exert abnormal functions in the sub-regions inside the pathway. A large number of genetic components within entire pathways presents a challenge for precise medical analyses (9–11). Subpathways are defined as local gene subregions within canonical biological pathways, and their dysfunction has been reported to be associated with the occurrence, development, and prognosis of cancer (12–14). Subpathways are also used as signature, biomarkers, and drug recognition of cancer (13, 15, 16).

The Metabolic dysfunction is an important cause of GBM. Hence, the study of the metabolic subpathway changes involved with GBM is of great significance to understanding the underlying mechanisms of disease and developing treatment strategies. The Kyoto Encyclopedia of Genes and Genomes (KEGG) database is one of the most commonly used databases in the world for storing high-quality biological metabolic pathway information (17). KEGG's metabolic pathway involves genes and metabolites to provide a basis for integrating the genome and metabolome. How to effectively use metabolic pathways and gene expression data to accurately identify metabolic abnormal subregions in GBM patients is a challenge.

Herein, we developed a method to accurately identify GBM-related metabolic subpathways by constructing a metabolic subpathway activity score matrix. According to metabolic pathway structure information provided by KEGG, we adopted the k-clique concept employed in social network analysis to define pivotal metabolic subpathways based on distance similarity among genes (12, 18). In order to more robust and flexible identify GBM-related metabolic subpathways, unsupervised Gene Set Variation Analysis (GSVA) methods were adopted to enrich the metabolic subpathways into ranked list of gene for each sample (19). At the same time, the use of multiple GBM data sets improved the stability and universality of subpathway recognition (13).

Through our method, 25 metabolic subpathways were identified as GBM-related metabolic subpathways. Survival analysis shows that gene *LSS* (lanosterol synthase) in metabolic subpathway 00100_6 has a significant impact on the survival of patients and some studies have proved that it may be a potential therapeutic target of GBM. (S)-2,3-Epoxysqualene, which is the substrate of the *LSS*, is located at a key position in the metabolic subpathway and has the potential to regulate the entire pathway. Therefore, we chose (S)-2,3-Epoxysqualene as the experimental target to regulate metabolic subpathway 00100_6 to verify the reliability of the GBM-related metabolic subpathway identified by our proposed method. Through the CCK8 experiment and wound-healing assay, we found that (S)-2,3-Epoxysqualene will be consistent with the activity and migration of GBM cells. Flow cytometry proved that (S)-2,3-Epoxysqualene can also inhibit the cycle of GBM cells and promote the apoptosis of GBM cells. These results indicate the reliability of our method for identifying disease-related metabolic subpathways based on the metabolic subpathway activity score matrix.

## Materials and Methods

### Excavating Metabolic Subpathways From the KEGG Database Using the k-clique Algorithm

We manually downloaded the corresponding XML files for all human metabolic pathways from the KEGG database. Each metabolic pathways in the KEGG database was converted into an undirected network diagram by connecting two genes (enzymes) into an edge if there is a common compound in their corresponding reactions. Based on the distance similarity between genes, we use the K-clique method in social network analysis to mine subpathways and the distance between all genes is no exceeding K (K = 4 is used) is determined as the metabolic subpathway. The R package crmSubpathway we developed can perform the subpathway mining process. In the crmSubpathway, subpathways can be mined by using the k-clique algorithm based on SubpathwayMiner package that we previously published. In the SubpathwayMiner, we have confirmed that the distance among all genes in mined subpathways decreases as the value of the parameter k reduces and the default value K = 4 can extract stable subpathways with consistent functions (20). Hence the crmSubpathway provides users with the default value of parameter k (k = 4). The setting of parameter k is flexible. Users can choose an appropriate parameter according to their needs.

### Constructing the Metabolic Subpathway Activity Score Matrix

The R package crmSubpathway is based on the GSVA method to construct a metabolic subpathway activity score matrix. The GSVA is a gene set enrichment (GSE) method that estimates variation of subpathway activity over a sample population in an unsupervised manner (19). In order to be able to process data in different formats of microarray and RNA-seq, kernel estimation of the cumulative density function (including Gaussian kernel and Poisson kernel) is used to evaluate the gene expression level in these data. Next, we assessed the enrichment score of metabolic subpathway, similar to the GSEA method using the Kolmogorov-Smirnov (KS)-like random walk statistic (21). We use the difference between the maximum and minimum enrichment scores as the final metabolic subpathway activity score. The metabolic subpathway activity score is a standard Gaussian distribution under the null hypothesis of no change in subpathway activity throughout the sample population.

### GBM Data Set and Difference Analysis of Subpathway Activity

We collected three independent GBM data sets totaling 427 patients from Sun et al. (22); Gravendeel et al. (23) and the cancer genome atlas (TCGA). The somatic mutation data, which is in the same batch as the gene expression data from the TCGA database, is also downloaded. Detailed information concerning the data is shown in Table 1 Probes containing zero values are deleted. Multiple probes correspond to one gene, and the average value of the probes is taken as the expression value of the gene. In order to fully display metabolic abnormalities, all GBM samples did not distinguish subtypes. These data were used to construct the subpathway activity score matrix; We then performed differential expression analysis on the subpathway activity scores using the limma package (http://www.bioconductor.org/packages/release/bioc/html/limma.html) (24). Finally, we took the intersection of the significantly different subpathways of these three data sets as GBM-related subpathways.

**Table 1:** Details of three previous GBM transcriptomic studies from the GEO repository and TCGA.

**Dataset ID**	**GBM**	**Normal**	**Experiment type**	**Contributors**	**Download**
GSE4290	77	23	Expression profiling by array	Sun et al. (22)	ftp://ftp.ncbi.nlm.nih.gov/geo/series/GSE4nnn/GSE4290/matrix/GSE4290_series_matrix.txt.gz
GSE16011	159	8	Expression profiling by array	Gravendeel et al. (23)	ftp://ftp.ncbi.nlm.nih.gov/geo/series/GSE16nnn/GSE16011/matrix/GSE16011_series_matrix.txt.gz
GDC TCGA Glioblastoma (GBM)	155	5	Expression profiling by RNA-seq	Genomic data commons	https://xenabrowser.net/datapages/?dataset=TCGA-GBM.htseq_fpkm.tsv&host=https%3A%2F%2Fgdc.xenahubs.net&removeHub=https%3A%2F%2Fxena.treehouse.gi.ucsc.edu%3A443

### Survival Analysis

We downloaded the clinical data corresponding to the samples in the TCGA GBM data set from the TCGA database (https://xenabrowser.net/datapages/?dataset=TCGA-GBM.survival.tsv&host=https%3A%2F%2Fgdc.xenahubs.net&removeHub=https%3A%2F%2Fxena.treehouse.gi.ucsc.edu%3A443). GBM samples were divided into high expression group and low expression group according to the median value of gene expression level in TCGA GBM data set. Subsequently, the single factor cox proportional-hazards model is used to calculate HR (hazard ratio) and the Kaplan-Meier method is used for survival analysis and the log rank test is used to assess significance. Survival analysis is implemented by the package survival (https://cran.r-project.org/web/packages/survival/index.html) and the survival curve is drawn by the package survminer (https://cran.r-project.org/web/packages/survminer/index.html).

### Cell Culture and Reagent Preparation

Human glioblastoma multiforme cell line U87-MG (Non-*IDH* mutation) was received from the College of Pharmacy, Daqing Branch of Harbin Medical University, and maintained in a humidified incubator at 37°C in a 5% CO_2_ atmosphere in Dulbecco's modified Eagle medium (DMEM, Gibco, Grand Island, NY, USA) supplemented with 10% fetal bovine serum (FBS, Gibco) and antibiotics (Gibco). (S)-2,3-Epoxysqualene used in all experiments was purchased from Sigma. The (S)-2,3-Epoxysqualene powder was dissolved in dimethyl sulfoxide (DMSO). The DMSO solution of (S)-2,3-Epoxysqualene and the culture solution (DMEM for the wound-healing assay) were further configured before use according to the usage concentration.

### Cell Viability Analysis

Cell viability was assayed using a Cell Counting Kit 8 (CCK8) (Dojindo, Japan), according to the manufacturer's instructions. U87-MG cells were seeded into 96-well plates at 5,000 cells per well for overnight incubation at 37°C. Then, U87-MG cells (500 μl per well) were treated with different concentrations of (S)-2,3-Epoxysqualene and cultured for 2, 4, 6, 8, or 12 h. Control cells were cultured in medium with the same volume of DMSO only. Ten microliters of CCK8 solution was added to each well before harvesting, and the optical absorption value at 450 nm was measured after 4 h incubation.

### Wound-Healing Motility Assay

A wound-healing assay was used to determine the ability of cell migration. Twelve hour serum-starved U87-MG cells (5 × 10^5^/well) were seeded into six-well plates and allowed to adhere for another 12 h. Confluent monolayer cells were scratched with a sterile 200-μl pipette tip, followed by PBS washing to remove debris and suspended cells. The experimental cells were cultured with the serum-free medium plus 100 nmol/L (S)-2,3-Epoxysqualene, and the control cells were incubated with serum-free medium only. The wounds were observed under a phase-contrast microscope at 0 and 12 h. Migration distance was calculated from the change in wound size during the 12 h period using Image J software.

### Flow Cytometric Analysis of the Cell Cycle and Apoptosis

Cell cycle analysis was performed using propidium iodide (PI) staining for DNA quantitation. Cells were harvested, washed, and centrifuged at 1,200 r/min for 5 min, and subsequently fixed in 70% ethanol at 4°C for >18 h, followed by washing with PBS. Cells were then resuspended in 500 μl PBS with 0.1 mg/mL DNase-free RNase A and 25 μg/mL PI and incubated for 30 min at 37°C in the dark. For cell cycle measurement, at least 2 × 10^4^ cells were collected and analyzed using a flow cytometer (FACSCalibur, BD, USA) and ModFit LT 3.2 (Verity Software House, Topsham, ME, USA).

Cell apoptosis was assessed by PI and Annexin V-FITC double staining. Collected cells were washed in 1 × binding buffer before resuspension to a cell density of 5 × 10^6^ cells/ml. The cells were then incubated in a staining solution containing 5 μl Annexin V-FITC and 5 μl PI (BD Sciences) for 15 min in the dark at room temperature according to the manufacturer's instructions. The analysis was conducted immediately using a flow cytometer.

### Statistics

All experiments were performed in triplicate. Data were analyzed using Origin 2018 statistical software and expressed as means ± SD. Heatmaps of the subpathway activity score matrix for each data set were generated with pheatmap in R (https://CRAN.R-project.org/package=pheatmap). K-means clustering and clustering maps are completed by factoextra package (https://cran.r-project.org/web/packages/factoextra/index.html). The pathway relationship network is drawn by Cytoscape_v3.6.1. Statistical comparisons between two groups were made using an unpaired Student's *t*-test, and *p*-values indicate significance as follows: ^*^p-value < 0.05, ^**^p-value < 0.01, ^***^p-value < 0.001.

## Results

### Identification of GBM-Related Metabolic Subpathways Based on the Subpathway Activity Score Matrix

**Figure 1:**
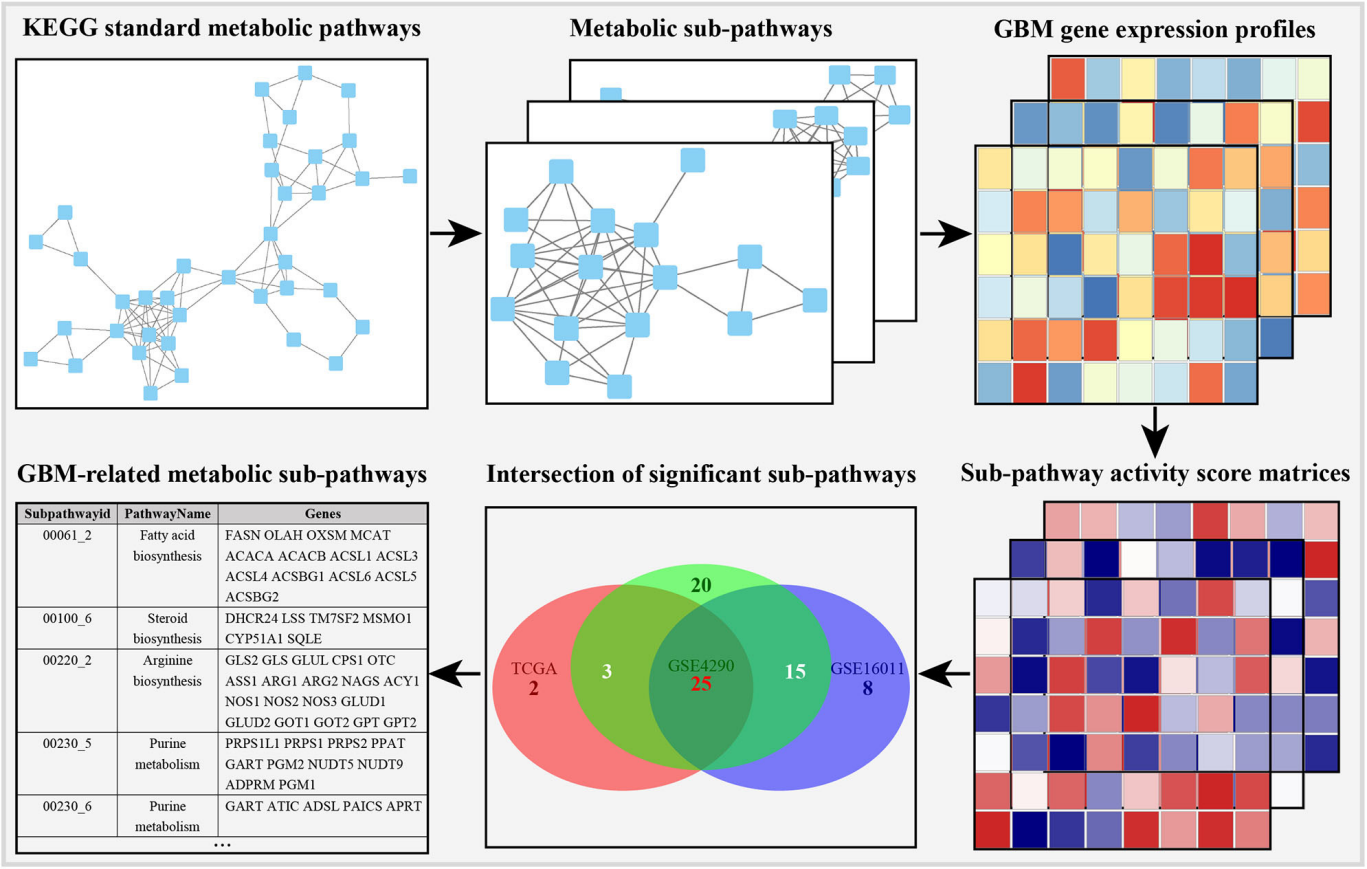
Outline of identification of GBM-related metabolic subpathways.

In order to obtain stable results, we selected the previous three GBM transcriptome studies based on the following criteria: (i) the data contain GBM and normal control samples, and the total number of samples is <30; (ii) different sequencing methods (DNA microarray or RNA-seq); (iii) the studies were conducted by independent groups. The details of the data are shown in Table 1. As shown in the metabolic subpathway analysis workflow (Figure 1), our study was mainly composed of three parts: (i) we first used the iSubpathwayMiner system to disassemble the KEGG metabolic pathway into connected metabolic subpathways based on the k-clique algorithm. The nodes of the metabolic subpathway represented one gene. We extracted the genes contained in each of the metabolic subpathways and constructed a list of metabolic subpathway gene sets. (ii) Then, three sets of gene expression profile data from the Gene Expression Omnibus (GEO) and TCGA databases were selected and input along with the list of metabolic subpathway gene sets into the GSVA R package to obtain three metabolic subpathway activity matrices. (iii) At last, for three metabolic subpathway activity matrices, we use the limma package to analyze the difference of metabolic subpathways between GBMs and healthy people and set adjusted *p*-value < 0.05 to determine significantly different metabolic subpathways.

**Figure 2:**
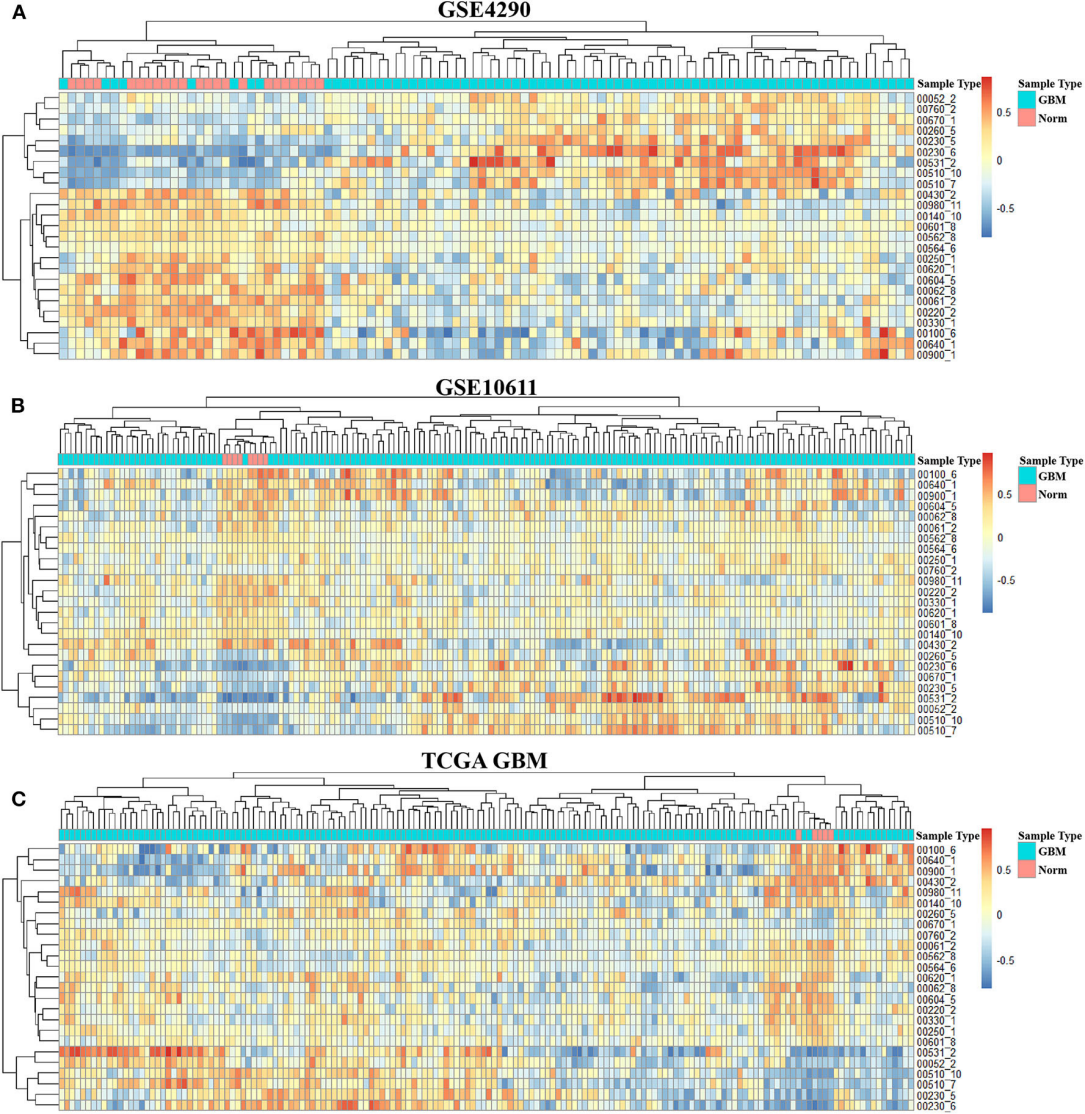
Activity score heat map of GBM-related metabolic subpathways in the activity score matrix of GSE4290 **(A)**, GSE10611 **(B)**, and GDC TCGA Glioblastoma **(C)**.

In order to integrate these data from different batches and sequencing methods, we use kernel estimation of the cumulative density function (kcdf) to assess the relative expression of genes in samples. Based on the ranking of genes in a single sample, we evaluated the activity of metabolic subpathways in each sample through gene enrichment. This can effectively reduce the batch effect between the metabolic subpathway matrix (Figure S1). In order to further obtain more stable results, we take the intersection of the significant subpathways of the three data set as GBM-related metabolic subpathways. This effort identified 25 significant subpathways as GBM-related metabolic subpathways. Detailed information on these subpathways is shown in Table S1. These subpathways showed significant differences in the heat map of the three subpathway activity score matrix (Figure 2).

### Analysis of GBM-Related Metabolic Subpathways

**Figure 3:**
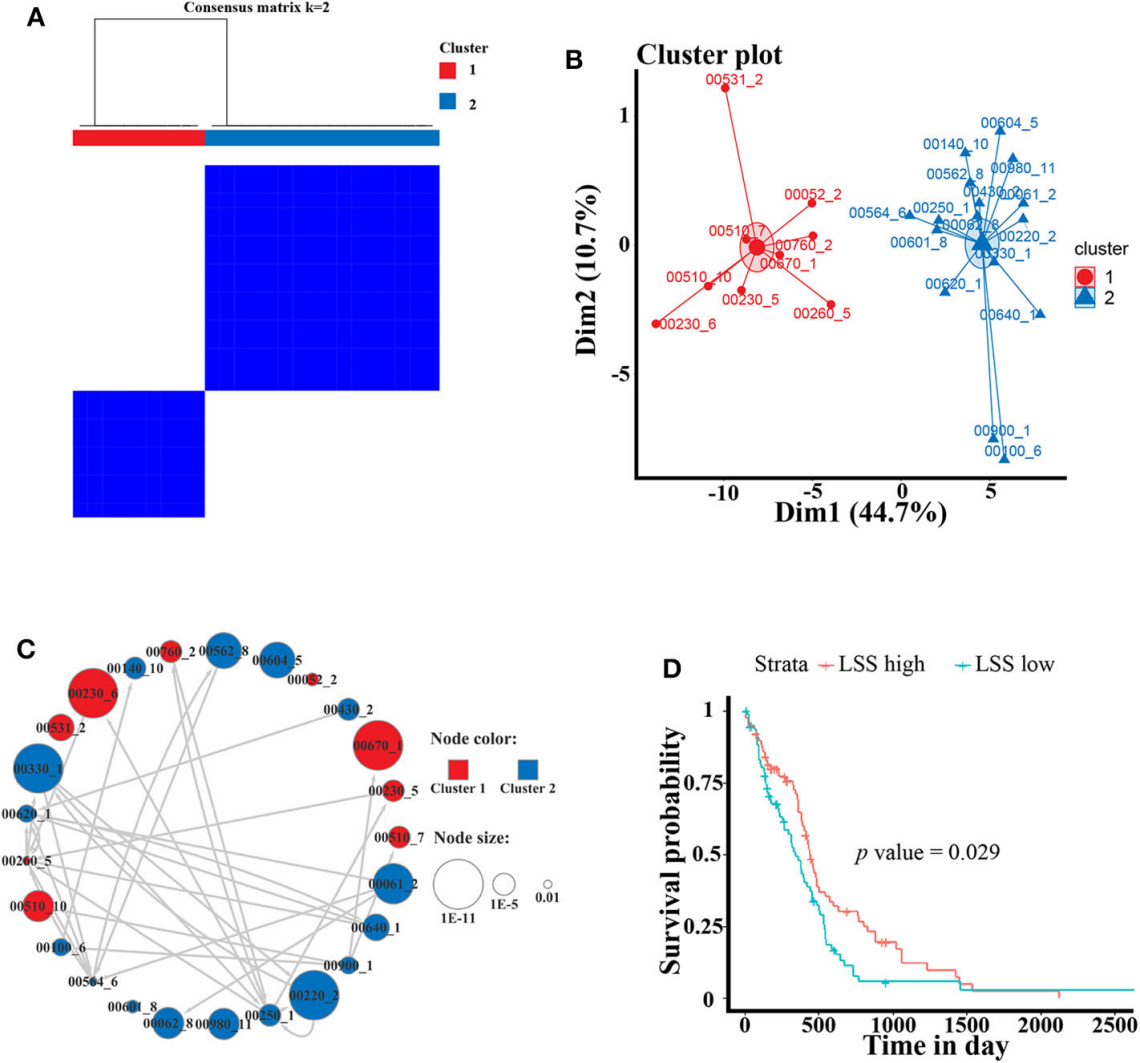
Cluster analysis of GBM-related metabolic subpathways and the survival analysis curve of key genes *LSS*. **(A)** Consistent clustering and **(B)** K-means clustering results of 25 GBM-related metabolic subpathways in GSE4290 data set. **(C)** The GBM-related metabolic subpathway relationship network map is manually drawn according to the functional relationship between the pathways in the KEGG database. The size of each node represents the significance of the difference in the activity scores of the sub pathways. The color represents the category of clustering. Arrows represent metabolic subpathway linked to other metabolic sub pathways and it is upstream of the linked metabolic subpathways. **(D)** Survival curve of the gene *LSS* of metabolic subpathway 00100_6 in TCGA GBM data set.

To better analyze the GBM-related metabolic subpathways, we use the subpathway activity matrix of all samples of the GSE4290 dataset to perform clustering analysis. The total within sum of square (wss), average silhouette width and gap statistics method are used to determine the optimal number of clusters of K-means clustering (Figures S2A–C). The area under cumulative distribution function (CDF) curve to evaluate the optimal number of clusters for consistent clustering (Figure S2D). These results indicate that the optimal number of clusters is two. The results of consistent clustering and K-means clustering are consistent (Figures 3A,B). As shown in Figure 2, hierarchical clustering of GBM-related metabolic subpathways in GSE4290 data set has consistent results and has good reproducibility in the GSE10611, TCGA GBM data set. This indicates that the 25 GBM-related metabolic subpathways identified by our method are highly robust.

The activity of the first class metabolic subpathway is up-regulated and the second class metabolic subpathway is down-regulation in GBM patients. We also manually plotted the relationship network diagram of these GBM-related metabolic subpathways according to the KEGG database. Although these subpathways have different degrees of misalignment in GBM, they are closely related (Figure 3C). The development of GBM cells may depend on the first class up-regulated metabolic subpathway. Studies show that the metabolic subpathways of first class contribute to the migration and invasion of GBM cells and it has an impact on the treatment and prognosis of GBM patients (25–31). There is a strong functional connection between subpathways of the second class (Figure 3C). A large number of studies have shown that some genes in the second class subpathway are signatures related to the survival of GBM patients or are targets for inhibiting GBM cell activity (32, 33). For example, studies have reported that *ACAT1* (cholesterol acyltransferase 1) in subpathways 00640_1 (propanoate metabolism) and 00900_1 (terpenoid backbone biosynthesis) may be a therapeutic target for the treatment of GBM (34). In addition, the low expression of *ABAT* (4-aminobutyrate aminotransferase) in subpathway 00640_1 is linked to poor survival (32). Although, downstream compounds of metabolic subpathway 00100_6 of the sterol biosynthesis pathway (such as cholesterol and steroid hormones) are closely related to GBM (33–35).

However, There is no study to elucidate the relationship between metabolic subpathway 00100_6 and GBM. We used the TCGA GBM data set and sample clinical data for survival analysis (see materials and methods for detailed procedures). The result of survival analysis shows that *LSS* in metabolic subpathway 00100_6 plays a significant role in the prognosis of GBM patients (HR = 1.5) (Figure 3D). *LSS* has also been proved to have a significant effect on GBM and may be a potential therapeutic target (36, 37). These results indicate that *LSS* may be a key gene of metabolic subpathway 00100_6. (S)-2,3-Epoxysqualene, which is the substrate of the *LSS*, is located at a key position in the metabolic subpathway and has the potential to regulate the entire pathway (Figure S3). In order to verify the reliability and accuracy of the GBM-related metabolic subpathway identification method we constructed and the effect of (S)-2,3-Epoxysqualene on GBM cells, we next conducted experiments with (S)-2,3-Epoxysqualene as the target to regulate metabolic subpathway 00100_6.

### (S)-2,3-Epoxysqualene Significantly Inhibited GBM Cell Viability and Migration

**Figure 4:**
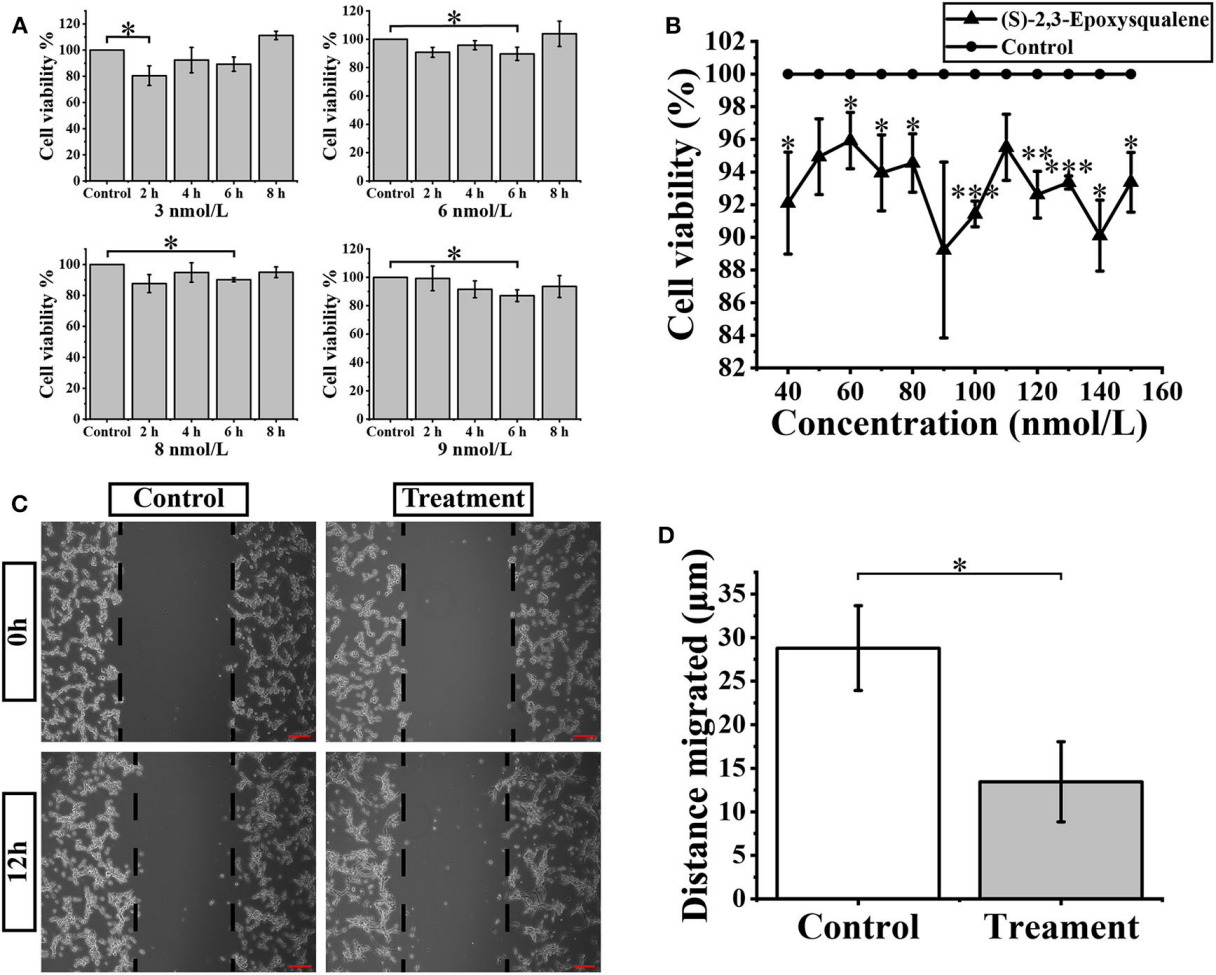
Cellular activity and migration analysis. **(A)** U87-MG cells were treated with the 3-9 nmol/L (S)-2,3-Epoxysqualene for 2–8 h, and cell viability was measured by the CCK8 assay. **(B)** CCK8 assay measures cell viability after treatment of 100 nmol/L (S)-2,3-Epoxysqualene of U87-MG cells for 12 h. **(C)** Effects of 100 nmol/L (S)-2,3-Epoxysqualene on U87 cell migration. The scale bar = 50 μm. **(D)** The migration distance of U87cells after 100 nmol/L (S)-2,3-Epoxysqualene treatment quantified by Image J software. Data represent means ± SD (*n* = 3). ^*^P < 0.05, ^**^P < 0.01, ^***^P < 0.001 by Student's *t* test.

(S)-2,3-Epoxysqualene is an intermediate compound of the steroid biosynthesis pathway. It is in the middle of subpathway 00100_6 and is also at the initial critical position of the steroid biosynthesis pathway. In our results for the metabolic subpathway analysis, subpathway 00100_6 was significantly down-regulated in GBM patients compared to healthy people. Therefore, we assumed that the metabolic subpathway 00100_6 would be activated by increasing the amount of (S)-2,3-Epoxysqualene to affect the GBM. A related study has shown that the inhibitory concentration of the inhibitor of the lanosterol cyclase downstream of (S)-2,3-Epoxysqualene is 10 nmol/L (38). Therefore, we selected a small concentration gradient of (S)-2,3-Epoxysqualene within 1-10 nmol/L to treat U87-MG cells, and we measured cell viability with CCk8 at 2, 4, 6, and 8 h (Figure 4A). We found that 3 nmol/L of (S)-2,3-Epoxysqualene had a significant inhibitory effect on GBM cells at 2 h of treatment. The inhibition effects of 6, 8, and 9 nmol/L at 6 h were the most significant. We also examined the effect of 5, 10, 20, and 30 nmol/L (S)-2,3-Epoxysqualene on GBM cell activity at 1–8 h. Although not significant, these concentrations have a strong tendency to inhibit cell viability at 4 h (Figure S4). However, this inhibitory effect time of (S)-2,3-Epoxysqualene on GBM cells is far from reaching the time when the cells begin to proliferate (39). In order to study the effects of (S)-2,3-Epoxysqualene on cell migration, cell cycle, and apoptosis of GBM, we tested the activity of higher concentrations of (S)-2,3-Epoxysqualene on GBM cells after 12 h of treatment (Figure 4B). We found that most of the concentrations of 40–150 nmol/L were significant after 12 h of treatment. Among these, 100 nmol/L (S)-2,3-Epoxysqualene had the most significant effect (*p* = 0.0002) on the activity of GBM and the inhibitory effect of (S)-2,3-Epoxysqualene on GBM cells has maintained a high significance after 100 nmol/L. Therefore, GBM cells cultured at 100 nmol/L (S)-2,3-Epoxysqualene for 12 h were used for subsequent experiments. In these results, we found that the inhibitory effect of (S)-2,3-Epoxysqualene on GBM cell activity is not stable, such as GBM cell activity increases at 4 h and then decreases at 6 h. However, this trend is consistent in 3, 6, 8, 9, and 30 nmol/L. This phenomenon indicate that GBM is more sensitive to (S)-2,3-Epoxysqualene and the trend of inhibition is reproducible.

We next performed a wound-healing assay using a 100 nmol/L (S)-2,3-Epoxysqualene treatment of GBM cells for 12 h. To limit the impact of cell growth on our wound-healing assay, we starved the cells before and during the wounding assay. The results showed that the migration distance of GBM cells was significantly reduced during 12 h of (S)-2,3-Epoxysqualene treatment (Figures 4C,D).

### Treatment With (S)-2,3-Epoxysqualene Induced GBM Cell Apoptosis and Cell Cycle

**Figure 5:**
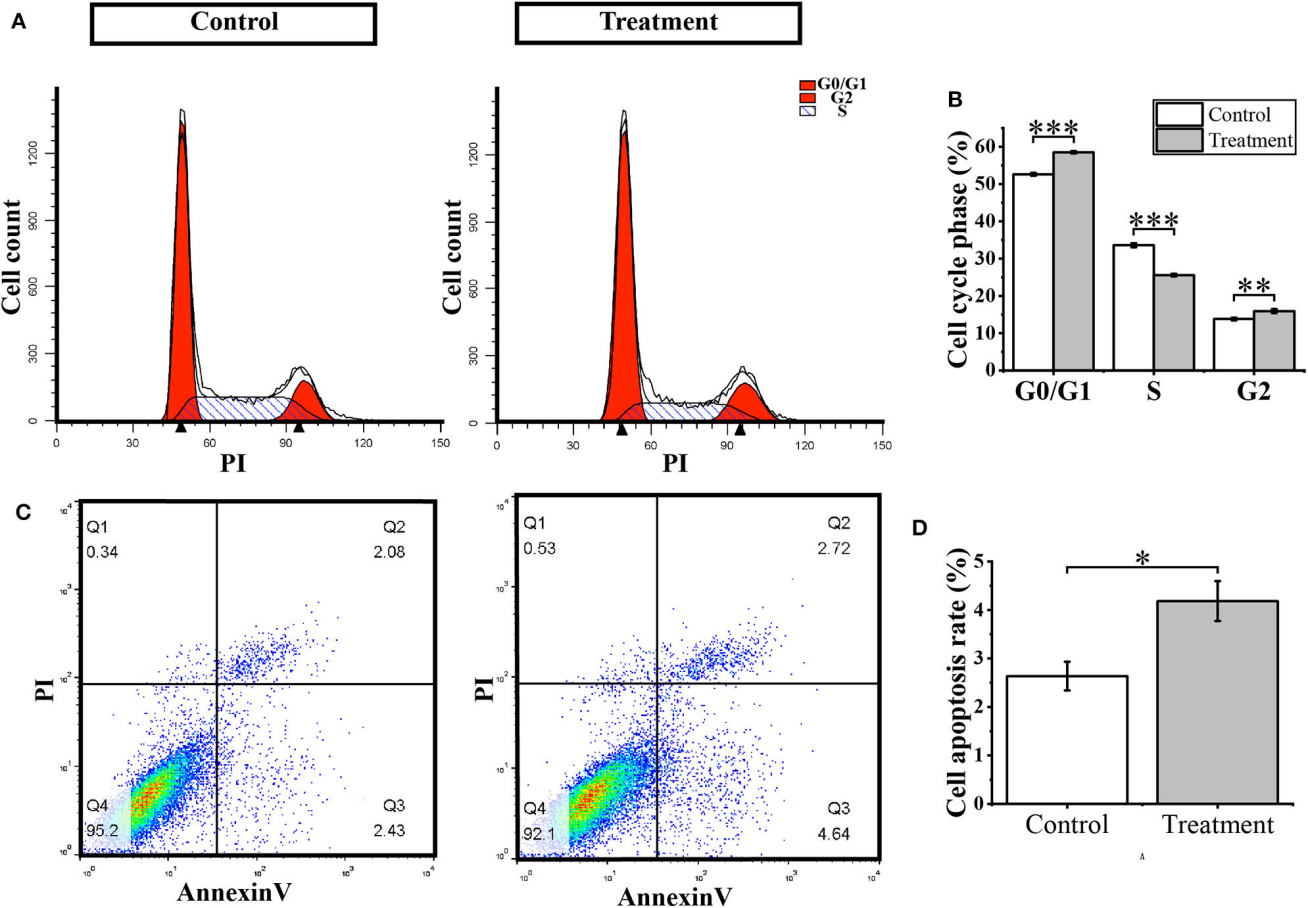
Cell cycle and apoptosis analysis. **(A)** Flow cytometry for cell cycle detection after treatment with 100 nmol/L (S)-2,3-Epoxysqualene U87 cells for 12 h. **(B)** Cell number distribution in each cell cycle. **(C)** Cell apoptosis detected by FACS analysis. **(D)** The ratio of apoptotic cells in U87 cells treated with 100 nmol/L (S)-2,3-Epoxysqualene for 12 h. Data represent means ± SD (*n* = 3). ^*^P < 0.05, ^**^P < 0.01, ^***^P < 0.001 by Student's *t*-test.

To further verify whether (S)-2,3-Epoxysqualene affects the cell cycle and apoptosis of GBM cells, we compared cell apoptosis and cell cycle between (S)-2,3-Epoxysqualene-treated and control GBM cells by flow cytometry. We also used 100 nmol/L (S)-2,3-Epoxysqualene to treat U87-MG cells and to detect apoptosis and cycle after 12 h. By counting the ratio of the number of cells in each cell cycle to the total number of cells, we found that U87-MG cells in the G0/G1 phase were accumulated in the (S)-2,3-Epoxysqualene-treated group, and there was a significant difference compared with the control group. The number of U87 cells in the S phase also decreased significantly after (S)-2,3-Epoxysqualene treatment (Figures 5A,B). This result indicates that (S)-2,3-Epoxysqualene can inhibit the proliferation of GBM cells by affecting the early stages of DNA synthesis (G0/G1). In addition, (S)-2,3-Epoxysqualene can also promote the apoptosis of U87-MG cells, and the apoptosis of U87-MG cells doubled compared with the control group (Figures 5C,D). Thus, treatment of (S)-2,3-Epoxysqualene can decrease cell numbers by inducing early apoptosis in U87-MG cells.

### Identify Metabolic Subpathways Associated With IDH Mutations in GBM

The metabolic subpathway identification method we constructed can be used not only for disease and normal but also for identifying metabolic subpathways related to specific phenotypes. Current research has confirmed that *IDH* (*IDH1* and *IDH2*) mutations have an important effect on the metabolic mechanism and prognosis of GBM patients (40, 41). Hence aiming to elucidate the metabolic sub-regions affected by *IDH* mutations, we downloaded the somatic mutation data from the TCGA database. According to the mutation status of *IDH*, GBM patients are divided into *IDH*-mutant GBMs and *IDH*-WT GBMs. Through the method we constructed and set *p* < 0.05, we identified 13 and 7 significant metabolic subpathways for *IDH*-mutant GBMs vs. Normal and *IDH*-mutant GBMs vs. *IDH*-WT GBMs, respectively. These significant metabolic subpathways are shown in Table S2. In our results, changes in fatty acid metabolism related subpathway 00062_9 (Fatty acid elongation) and 00071_1 (Fatty acid degradation) between *IDH* mutations and normal healthy person may contribute to the tumorigenesis of gliomas by priming cells for growth (42, 43). *ALDH6A1* (Aldehyde dehydrogenase 6), which is involved in metabolic subpathway 00280_7 (Valine, leucine and isoleucine degradation), 00640_4, and 00640_5 (Propanoate metabolism), has a significant change in abundance in relation to the *IDH1* mutation (44, 45). Moreover, *BCAT1* (branched-chain amino acid transaminase 1) in metabolic subpathway 00280_2 is only expressed in *IDH* wild-type tumors. *BCAT1* expression could be suppressed by ectopic overexpression of mutant *IDH1* in immortalized human astrocytes and suppression of *BCAT1* significantly reduce tumor growth in a glioblastoma xenograft model (46). For the significant metabolic pathways of *IDH*-mutant GBMs vs. Normal and *IDH*-mutant GBMs vs. *IDH*-WT GBMs, the metabolic subpathway 00630_1 (Glyoxylate and dicarboxylate metabolism) and 00120_11 (Primary bile acid biosynthesis) are mined by taking the intersection. The areas of these two metabolic subpathways in the KEGG pathway diagram are shown in Figures S5, S6. The study has confirmed that the gene *ACOX2* (acyl-CoA oxidase 2) in subpathway 00120_11 and *GLDC* (glycine decarboxylase), *SHMT 1/2* (serine hydroxymethyltransferase 1/2) of 00630_1 are closely related to *IDH* mutation in GBM (47–49). These results indicate that metabolic subpathways 00120_11 and 00260_1 are key regulatory targets for *IDH* mutations.

## Discussion

Metabolic changes are an important hallmark of cancer. At present, the analysis of GBM metabolic changes mainly depends on basic experimental means. However, the emergence of high-throughput sequencing data provides a basis for a more comprehensive understanding of the metabolic changes involved in GBM. Herein, we integrated gene expression data with the metabolic subpathways from the KEGG database to construct a metabolic subpathway activity score matrix and mine all GBM-related metabolic subpathways. Using this method, we found that (S)-2,3-Epoxysqualene as a potential therapeutic target of GBM could inhibit cell activity, migration, and proliferation and that it could promote apoptosis of U87-GM cells.

In order to better identify the abnormal metabolic pathways of GBM, we developed a method based on the metabolic subpathway activity score matrix. By analyzing three GBM gene expression data sets from the TCGA and GEO databases, our method identified 25 GBM-related metabolic subpathways. Through the K-means clustering method, these subpathways were clustered into three categories. The first class of metabolic subpathway activity was significantly upregulated in GBM, probably because GBM cells need these metabolites. Likewise, previous research indicates that serine and glycine, which belong to our first class of subpathways, are essential precursors of proteins, nucleic acids, and lipids involved in cancer growth and that they contribute significantly to the energy requirements of cancer cells. In some cancers, an increase in serine synthesis is used as a marker of poor prognosis (50, 51). Similarly, Wang et al. have also confirmed that mitochondrial serine hydroxymethyltransferase 2 (*SHMT2*) in serine and glycine metabolism is overexpressed in gliomas and promotes tumor cell proliferation (52). In addition, the metabolic subpathways 00510_7/00510_10, 00531_2, and 00760_2 belonging to the respective pathways N-Glycan biosynthesis, Glycosaminoglycan degradation, and Nicotinate and nicotinamide metabolism, have been confirmed to be closely related to the migration and invasion of GBM (25–28). The *MTHFD2* (methylenetetrahydrofolate dehydrogenase (NADP+ dependent) 2) gene in the metabolic subpathway 00670_1 of the one carbon pool by folate pathway is one of the features of GBM prognosis by survival analysis and the random survival forest algorithm (29). Elevated expression of purine synthetic enzymes (*PRPS1, ADSL*, and *GMPS*) in metabolic subpathway 00230_5/00230_6 of the purine metabolic pathway is correlated with poor prognosis in glioblastoma patients (30). Azacoccone E, an aza-epicoccone derivative from the culture of Aspergillus flavipes, is used in treatment for GBM by inhibiting *PHGDH* (3-phosphoglycerate dehydrogenase) in the metabolic subpathway 00260_5 (31). Metabolic subpathway 00052_2, which belongs to the pathway of galactose metabolism, has been shown to be involved in galactose-specific C-type lectin receptor stimulant immunotherapy of an experimental glioma (53).

The activities of the second types of metabolic subpathways are down-regulated in GBM. This may be because these metabolic processes or some genes in the metabolic subpathway are not conducive to the maintenance and growth of GBM cells. Similar to our results, the arginine and 2-Oxoarginine signal intensities in the second class of metabolic subpathway 00330_1 belonging to the arginine and proline metabolism pathways are inversely related to the risk of glioma (54). The enzyme *ABAT* in the second class of metabolic subpathway 00640_1 demonstrated that its low expression in GBM patients is associated with poor survival (32). The expression levels of genes *ASS1* (arginosuccinate synthase 1), *ASPA* (aspartoacylase), *GOT1* (glutamic-oxaloacetic transaminase 1), *GAD1* (glutamate decarboxylase 1), *GAD2* (glutamate decarboxylase 2) and *ABAT* in the metabolic subpathways 00250_1 (alanine, aspartate and glutamate metabolism), 00330_1 (arginine and proline metabolism), 00220_2 (arginine biosynthesis), and 00430_2 (taurine and hypotaurine metabolism) will affect the survival of GBM patients (32). These results indicate the reliability of the method we constructed to identify disease-related metabolic subpathways.

The metabolic subpathway 00100_6 in the second class belongs to the sterol biosynthesis pathway. No related studies have shown that it is related to the occurrence and development of GBM. However, previous research has established that downstream cholesterol and sterol hormones regulated by 00100_6 will affect the progression of GBM (35, 55–58). In addition, vitamin D metabolism is directly regulated by calcidiol 1-monooxygenase (*CYP27B1*) and vitamin D 1,25-hydroxylase (*CYP105A1*) downstream of the metabolic subpathway 00100_6, and studies have shown that the level of 25-hydroxyvitamin D (25(OH)D) in the serum of intermediates of vitamin D metabolism is opposite to that of patients with GBM-specific subtypes (59). Similar results were also confirmed in the study of Ida Emanuelsson et al. experiments with T98G Human Glioblastoma Cells treated with vitamin D analogs tacalcitol and calcipotriol showed that vitamin D inhibits the proliferation and migration of GBM cells (60). Hence, we inferred that the metabolic subpathway 00100_6 may be a potential therapeutic target for GBM. (S)-2,3-Epoxysqualene is located in the central region of the subpathway and the gene *LSS* downstream of (S)-2,3-Epoxysqualene has a significant impact on the survival of GBM patients, suggesting a greater impact on the entire subpathway and a potential high association with GBM. Through the CCK8 experiment, we found the inhibitory effect of (S)-2,3-Epoxysqualene on GBM cells was fluctuating. However, volatility trend is consistent at 3, 6, 8, 9, and 30 nmol/L. This phenomenon has high reproducibility. In addition, after 12 h of treatment, 100 nmol/L (S)-2,3-Epoxysqualene had the most significant effect on the activity of GBM and the inhibitory effect of (S)-2,3-Epoxysqualene on GBM cells has maintained a high significance after 100 nmol/L. This results of CCK8 also verified that (S)-2,3-Epoxysqualene significantly inhibited the activity of U87-GM cells, with small-scale short-term or large-dose long-term effects (Figures 4A,B). We then used flow cytometry to find that (S)-2,3-Epoxysqualene inhibits GBM cell activity by promoting apoptosis and inhibiting the cell cycle. Further, the wound-healing motility assay found that the migration distance of cells after (S)-2,3-Epoxysqualene treatment was significantly shorter than that of the control group. These experimental results not only indicate that (S)-2,3-Epoxysqualene can effectively inhibit the activity, amplification, and migration of GBM cells but further verify the reliability of our method.

Therefore, according to our current research, we can infer that the therapeutic mechanism of (S)-2,3-Epoxysqualene on GBM is to affect downstream sterols and steroid hormones. These results also prove the reliability of the metabolic subpathway identification method we constructed.

Our method creatively combined gene expression profile data with metabolic subpathways by constructing a metabolic subpathway activity score matrix to elevate research levels from genes to gene sets. As reported by Wenhua Lv et al., even the smallest pathways have tens to hundreds of bases. The traditional pathway identification methods do not accurately consider the combined effect of the interesting molecules and neglects expression correlations or topological features embedded in the pathways (11). Some studies suggest that the functional similarity between two genes increases as their distance in pathways decreases (61, 62). In social network analysis, a k-clique in a graph is considered as a sub-graph where the distance between any two nodes is no >k (63). Li et. al. proposed that it is appropriate to mine subpathway in the pathway networks with k = 4 and they have demonstrated that “4-clique” method could effectively identify risk subpathways associated complex diseases (20). Hence, we extracted the metabolic subpathway data from the KEGG database using the k-clique algorithm to make up for the defects of single-gene analysis and to consider gene interactions. Our method for constructing a metabolic subpathway activity score matrix is based on the GSVA method. The Gaussian kernel and discrete Poisson kernel methods provided by the GSVA method enable us to process gene sequencing data from different sources (DNA microarray and RNA-seq) (19). To make the results more stable, we selected the GBM gene expression datasets from the TCGA and GEO databases. These three data sets were obtained by different high-throughput sequencing techniques, including microarray and RNA-seq, and we considered the intersection of the significant metabolic subpathways of these three data as GBM-related metabolic subpathways. Moreover, the metabolic subpathway identification method can be used not only for disease and normal but also for identifying metabolic subpathways related to specific phenotypes. Herein, we also identified metabolic subpathways related to *IDH* mutations in GBM. Compared with traditional methods based on basic biological experiments on the metabolic mechanism of GBM, our method not only shortens the research cycle but also displays a robust and accurate performance in more completely identifying the metabolic changes involved in GBM.

Our study has several limitations: First, our study is based on high-throughput sequencing data and KEGG metabolic pathway data from GBM patients. We have not integrated more data sets, as for example the GBM dataset in the Chinese Glioma Genome Atlas (CCGA) database or the metabolic gene set in the Gene Ontology (GO) database. A more comprehensive description of the metabolic mechanisms of GBM remains to be found in the development of these databases. In another aspect, the study of (S)-2,3-Epoxysqualene's therapeutic effects on GBM was performed at the cell line level. However, our data for GBM metabolic subpathway analysis are the sequencing results of human tissue samples, and we have integrated three data sets to compensate for the lack of cell-based analyses. In addition, our constructed metabolic pathway activity score matrix contains sample information, which provides the possibility to combine subpathways with samples to identify biomarkers of specific disease subgroups and their associated metabolic subpathways. As a next step, we will integrate more gene regulatory mechanisms such as DNA methylation and micRNA to more deeply investigate disease abnormalities.

In conclusion, we have developed a new method for stable and accurate identification of GBM-associated metabolic subpathways. The subpathway 00100_6 of the sterol synthesis pathway was determined to be abnormal in GBM patients. The experimental results also showed that the regulation of subpathway 00100_6 by targeting (S)-2,3-Epoxysqualene could affect the activity, proliferation, and apoptosis of GBM cells to achieve antitumor effects. All of these results indicate that our method provides an accurate and reliable approach for the identification of metabolic abnormalities associated with GBM or other diseases, and the method provides effective guidance for clinical studies of disease treatment and metabolic pathogenesis. We also identified subpathway 00100_6 as a novel target for GBM treatment, and (S)-2,3-Epoxysqualene may be used as a drug for the treatment of GBM.

## Data Availability Statement

The datasets generated for this study can be found in the GEO database: GSE4290; GSE16011. TCGA database: https://xenabrowser.net/datapages/?dataset=TCGA-GBM.htseq_fpkm.tsv&host=https%3A%2F%2Fgdc.xenahubs.net&removeHub=https%3A%2F%2Fxena.treehouse.gi.ucsc.edu%3A443.

## Author Contributions

QK and JH designed the research. XH performed the research and wrote the manuscript. DW acquired the data and reviewed and edited the manuscript. PZ, CL, LC, and YH analyzed the data JZ, WZ, LM, JW, and HL contributed materials or analytic tools. All authors contributed to the article and approved the submitted version.

## Conflict of Interest

The authors declare that the research was conducted in the absence of any commercial or financial relationships that could be construed as a potential conflict of interest.
