# Supplementary material for: Inference of Subpathway Activity Profiles Reveals Metabolism Abnormal Subpathway Regions in Glioblastoma Multiforme
Source: Front Oncol. 2020 Sep 11;10:1549. doi: 10.3389/fonc.2020.01549 (PMC7533644; doi:10.3389/fonc.2020.01549)
Supplement: Supplementary file 3 [file Data_Sheet_1.docx]

Supplementary Material


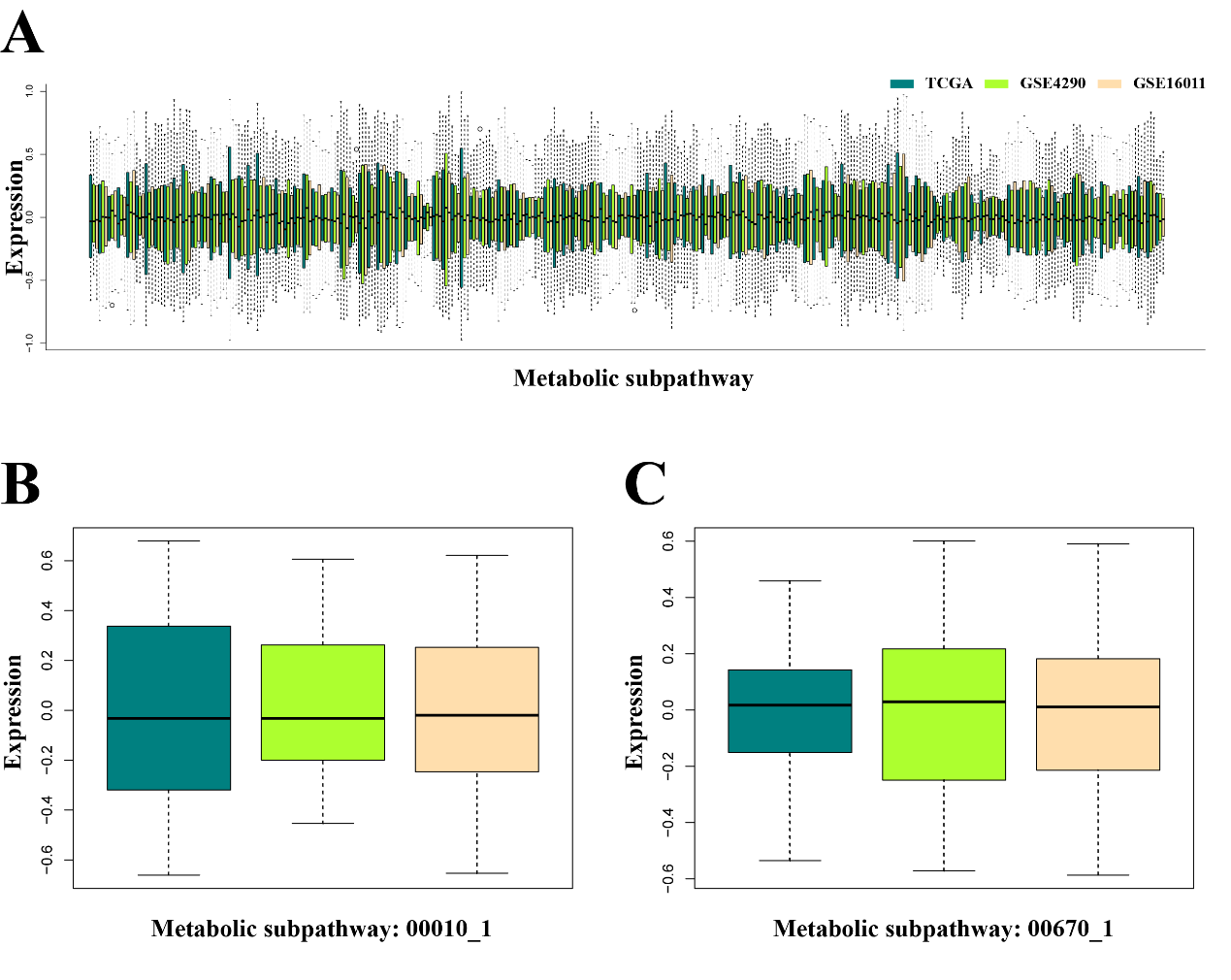


**Figure S1.** The box plot of expression of metabolic subpathways in TCGA GBM, GSE4290 and GSE16011 data set. (A) The expression distribution of each metabolic subpathway in all samples of three data sets. (B-C) Two randomly selected metabolic subpathways are displayed.


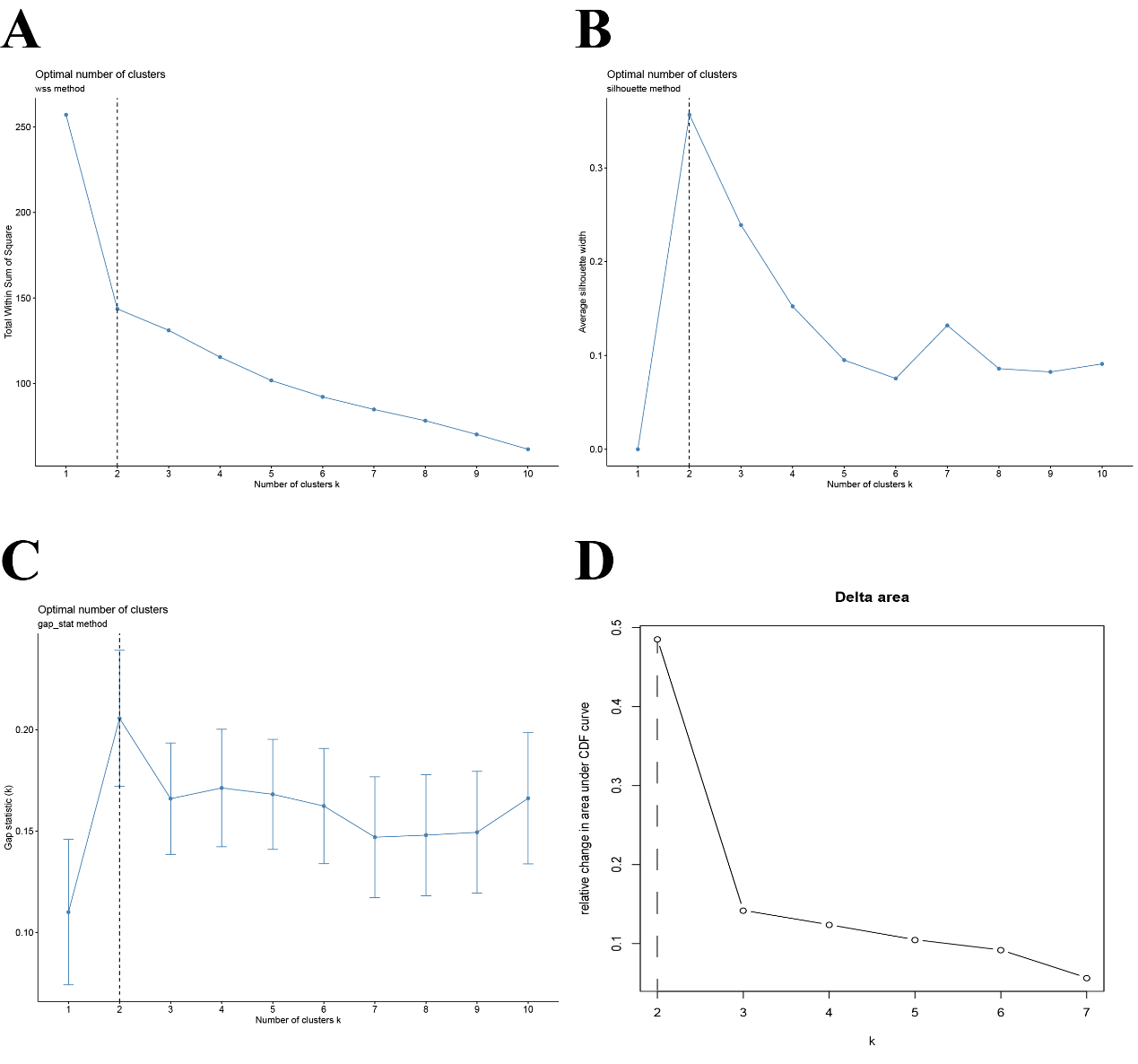


**Figure S2.** Evaluate the number of clusters in the cluster. (A) The total within sum of square (wss), (B) average silhouette width and (C) gap statistics method evaluates the optimal number of clusters of K-means clustering. (D) The area under cumulative distribution function (CDF) curve to evaluate the optimal number of clusters for consistent clustering.


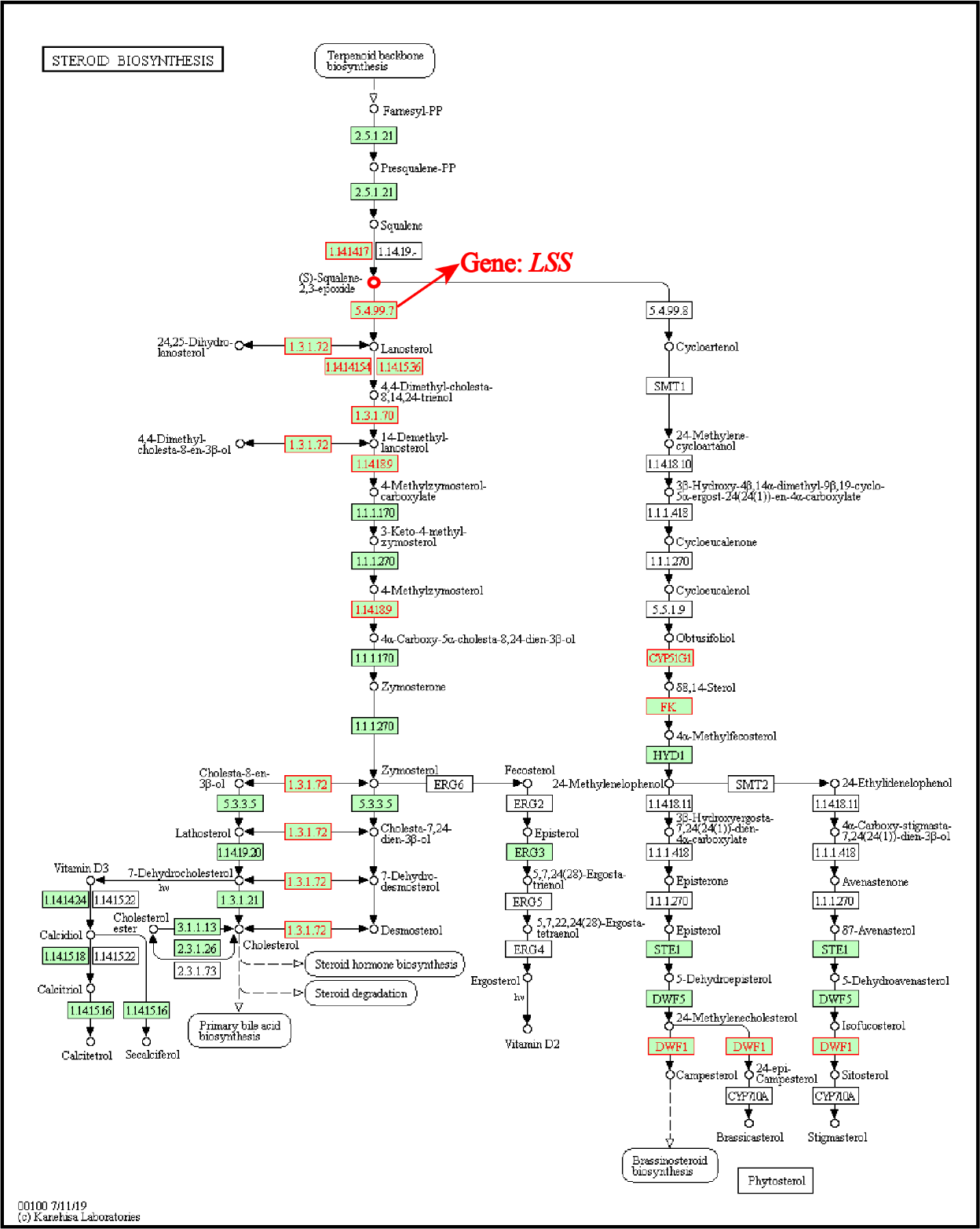


**Figure S3.** The ‘Steroid biosynthesis’ pathway. The nodes of the red matrix belong to the subpathway 00100_6 region. The node in the red circle is (S)-2,3-Epoxysqualene in the key position of the subpathway.

**
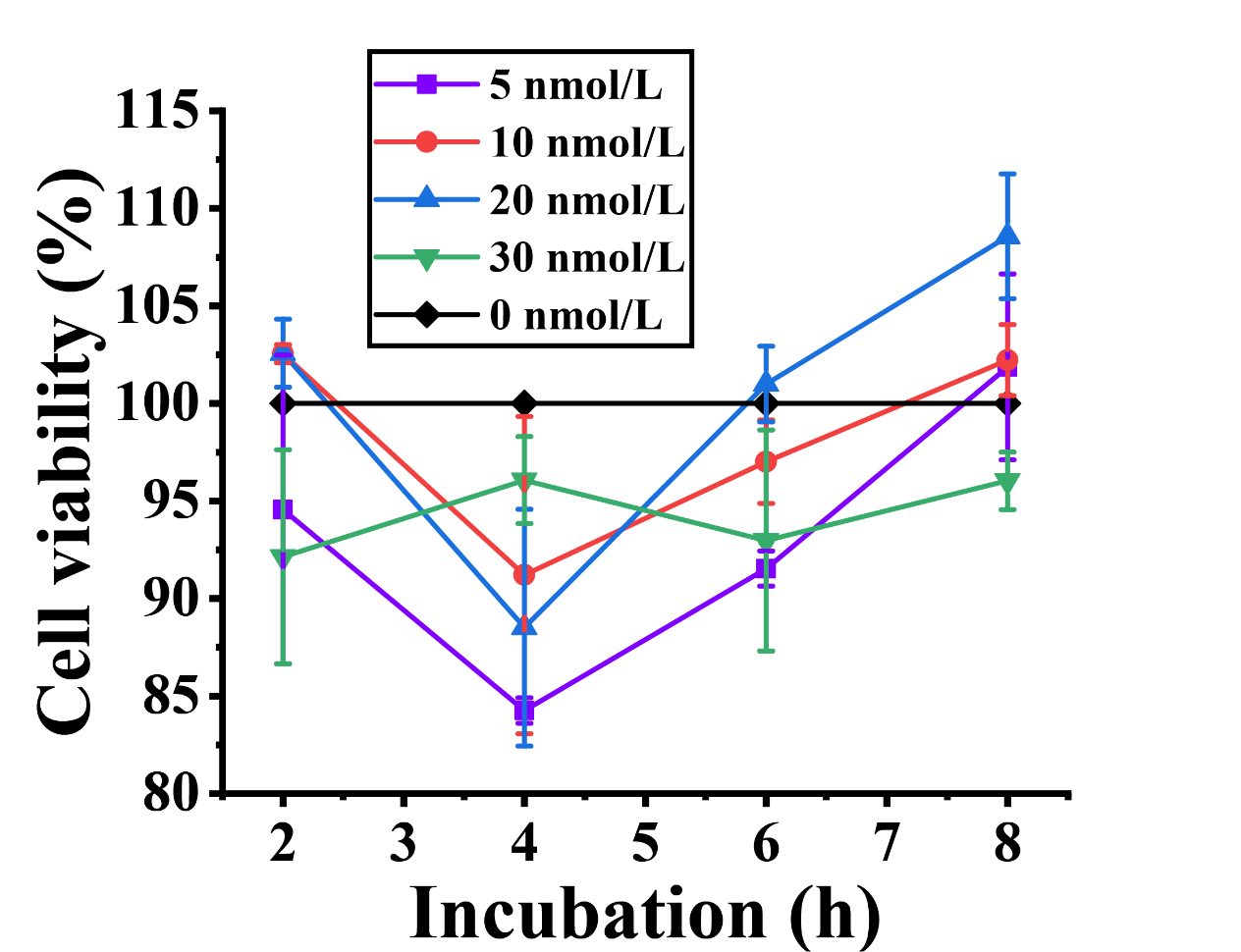
**

**Figure S4.** U87-MG cells were treated with 5-30 nmol/L of (S)-2,3-Epoxysqualene for 2-8 h. The cell viability was measured by CCK8 assay.


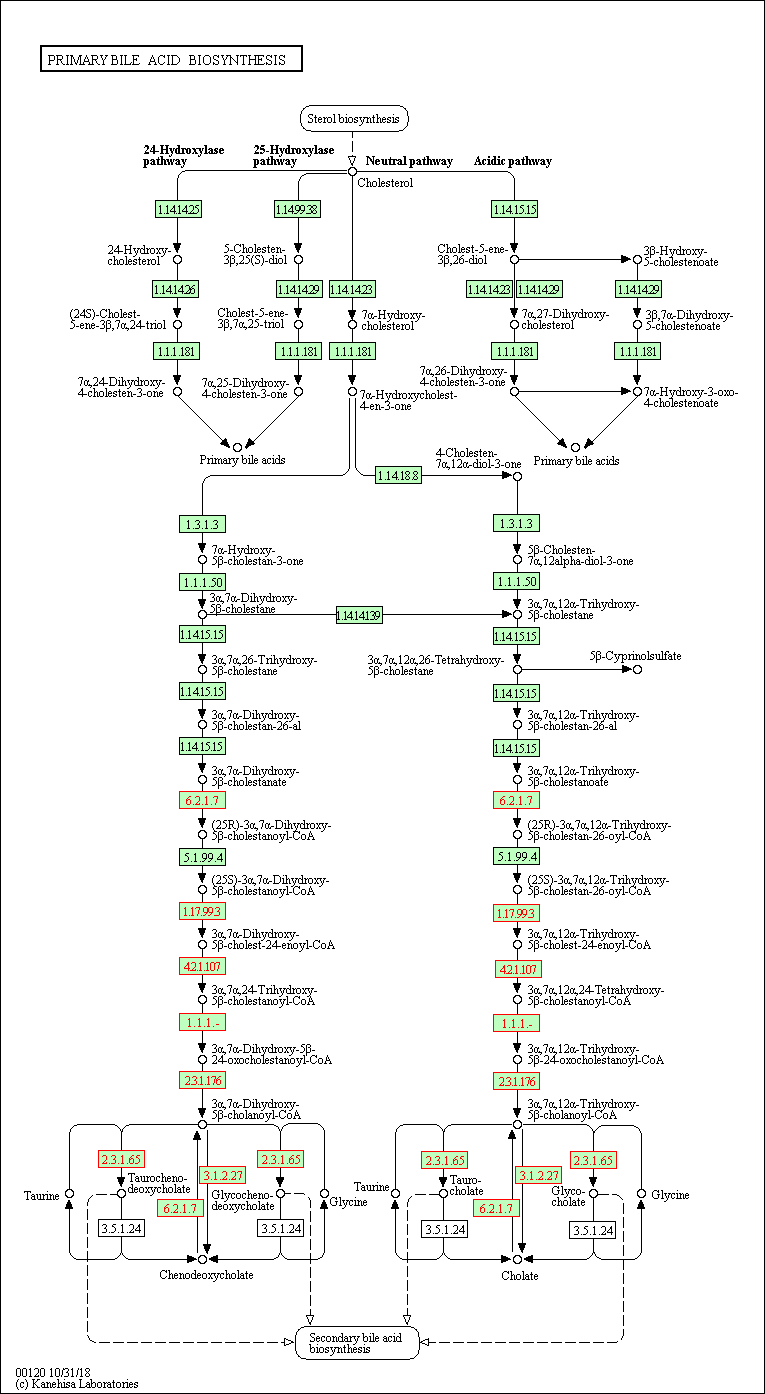


**Figure S5.** The ‘Primary bile acid biosynthesis’ pathway. The nodes of the red matrix belong to the subpathway 00120_11 region.


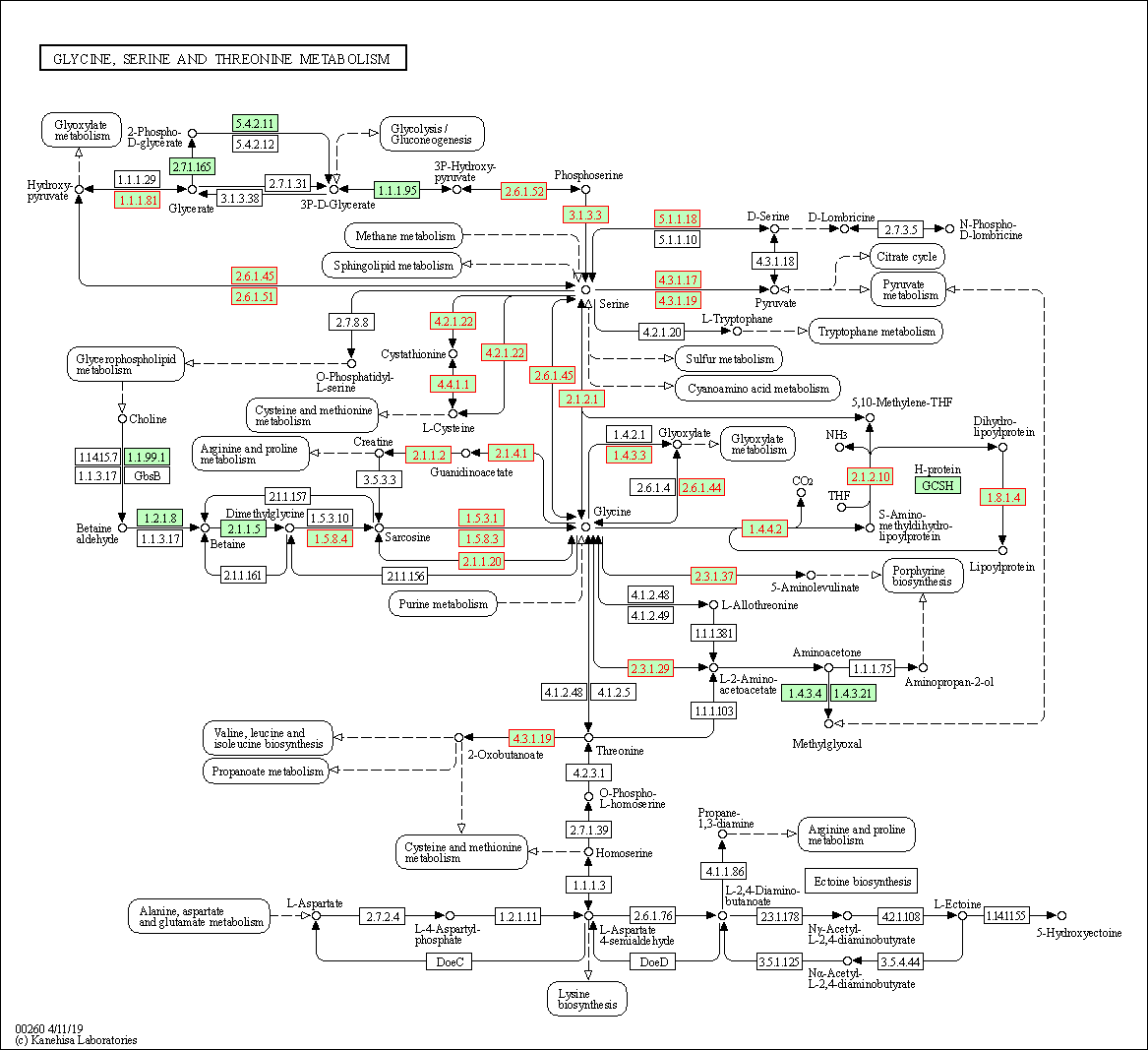


**Figure S6.** The ‘Glycine, serine and threonine metabolism’ pathway. The nodes of the red matrix belong to the subpathway 00260_1 region.
